# Genetic Deletion of mGlu3 Metabotropic Glutamate Receptors Amplifies Ischemic Brain Damage and Associated Neuroinflammation in Mice

**DOI:** 10.3389/fneur.2021.668877

**Published:** 2021-06-17

**Authors:** Federica Mastroiacovo, Manuela Zinni, Giada Mascio, Valeria Bruno, Giuseppe Battaglia, Julien Pansiot, Tiziana Imbriglio, Jerome Mairesse, Olivier Baud, Ferdinando Nicoletti

**Affiliations:** ^1^Department of Molecular Pathology, I.R.C.C.S. Neuromed, Pozzilli, Italy; ^2^Inserm UMR1141 NeuroDiderot, University of Paris Diderot, Sorbonne Paris Cité, Paris, France; ^3^Department of Physiology and Pharmacology, Sapienza University of Rome, Rome, Italy; ^4^Laboratory of Child Growth and Development, University of Geneva, Geneva, Switzerland; ^5^Division of Neonatology and Pediatric Intensive Care, Children's University Hospital of Geneva, Geneva, Switzerland

**Keywords:** focal ischemia, knockout mice, neuroinflammation, pro-inflammatory genes, mGlu3 receptors

## Abstract

**Backgroud:** Type-3 metabotropic glutamate (mGlu3) receptors are found in both neurons and glial cells and regulate synaptic transmission, astrocyte function, and microglial reactivity. Here we show that the genetic deletion of mGlu3 receptors amplifies ischemic brain damage and associated neuroinflammation in adult mice. An increased infarct size was observed in mGlu3^−/−^ mice of both CD1 and C57Black strains 24 h following a permanent occlusion of the middle cerebral artery (MCA) as compared to their respective wild-type (mGlu3^+/+^ mice) counterparts. Increases in the expression of selected pro-inflammatory genes including those encoding interleukin-1β, type-2 cycloxygenase, tumor necrosis factor-α, CD86, and interleukin-6 were more prominent in the peri-infarct region of mGlu3^−/−^ mice. In contrast, the expression of two genes associated with the anti-inflammatory phenotype of microglia (those encoding the mannose-1-phosphate receptor and the α-subunit of interleukin-4 receptor) and the gene encoding the neuroprotective factor, glial cell line-derived neurotrophic factor, was enhanced in the peri-infarct region of wild-type mice, but not mGlu3^−/−^ mice, following MCA occlusion. In C57Black mice, the genetic deletion of mGlu3 receptors worsened the defect in the paw placement test as assessed in the contralateral forepaw at short times (4 h) following MCA occlusion. These findings suggest that mGlu3 receptors are protective against ischemic brain damage and support the way to the use of selective mGlu3 receptor agonists or positive allosteric modulators in experimental animal models of ischemic stroke.

## Introduction

Ischemic stroke is the second cause of mortality and disability worldwide due to the lack of effective therapies besides intravenous thrombolysis, which is restricted to selected patients and useful only in a short therapeutic window. Understanding the mechanisms underlying neuronal vulnerability to ischemic damage may pave the way to novel therapeutic strategies in cerebrovascular disorders. In the area close to the occluded vessel, two distinct zones can be distinguished: the core (a zone of severe ischemia and neuronal death) and the penumbra (a zone of moderate ischemia) (1). Up to few hours following the vessel occlusion, the size of both areas is almost equivalent (2); however, as the ischemic injury progresses, the penumbra becomes the main affected area (3), in which the neurons are still salvageable with appropriate interventions. Excitotoxicity, oxidative stress, neuroinflammation, mitochondrial damage, and lack of neuroprotective factors are the established mechanisms of ischemic neuronal damage (4–8). These mechanisms are shaped by metabotropic glutamate (mGlu) receptors, which are G-protein coupled receptors activated by glutamate.

mGlu receptors form a family of eight subtypes, subdivided into three groups on the basis of their primary sequence homology and transduction mechanisms. Group I comprises mGlu1 and mGlu5 receptors, which are coupled to G_q/11_ proteins. Their activation leads to phosphatidylinositol-4,5-bisphosphate hydrolysis, with ensuing formation of inositol-1,4-5-trisphosphate and diacylglycerol. Group II (mGlu2 and mGlu3) and group III (mGlu4, -6, -7, and -8) receptors are coupled to G_i/o_ proteins in heterologous expression systems (9). Most of these subtypes are found in neurons, astrocytes, and microglia and regulate many features of the tetrapartite synapse formed by presynaptic terminals, postsynaptic elements, and surrounding astrocytes and microglia.

Early studies of mGlu2 and mGlu3 receptors (10, 11) did not differentiate between the two subtypes because of the lack of selectivity of orthosteric agonists and the belief that the function of the two receptors was similar. Using both genetic and subtype-selective pharmacological tools, it is now clear that mGlu2 and mGlu3 receptors are functionally different and may have an opposite impact on the mechanisms of neurodegeneration/neuroprotection. While both subtypes are presynaptically localized and negatively modulate neurotransmitter release, mGlu3 receptors are also found in post-synaptic elements, and their activation boosts mGlu5 receptor signaling (12, 13). In addition, activation of mGlu2 receptors drives microglial cells toward a pro-inflammatory/neurotoxic phenotype (14, 15), whereas activation of mGlu3 receptors induces an anti-inflammatory phenotype (16). In addition, mGlu3 receptors (but not mGlu2 receptors) are present in astrocytes, and their activation stimulates the production of neurotrophic factors (17, 18). Using the four-vessel occlusion model of transient global ischemia in rats, we have shown that selective pharmacological activation of mGlu2 receptors amplified hippocampal damage, whereas mGlu2 receptor blockade was neuroprotective (19). Similar findings were obtained using mice lacking mGlu2 receptors, in which the infarct size was enhanced in response to transient focal ischemia (20). To our knowledge, there are no studies on mGlu3 receptors and brain ischemia, although the selective activation of mGlu3 receptors was shown to be neuroprotective in *in vitro* studies (17, 21–24).

Here we used two different strains of mice to examine whether the genetic deletion of mGlu3 receptors affects brain damage and associated neuroinflammation induced by the permanent occlusion of the middle cerebral artery (MCA).

## Materials and Methods

### Animals

Wild-type mice (mGlu3^+/+^) or mGlu3^−/−^ mice on C57Black genetic background (22) and wild-type mice (mGlu3^+/+^) or mGlu3^−/−^ mice on CD1 genetic background (25) were generated by homozygous breeding (in-house production). Adult male mice weighing 25 g were housed under controlled conditions (ambient temperature, 22°C; humidity, 40%) on a 12-h light–dark cycle with food and water *ad libitum*. Studies were performed in agreement with the national and international guidelines and regulations on animal care and use and were approved by the Neuromed Institutional Animal Care and Use Committee and by the Italian Ministry of Health. All efforts were made to minimize animal suffering and to reduce the number of animals used.

### Permanent Focal Ischemia in Mice

Permanent focal cerebral ischemia was induced by distal electrocauterization of the MCA (26–28). The mice were anesthetized with chloral hydrate (400 mg/kg, i.p.), and an incision was made between the outer canthus of the eye and the external auditory meatus. The temporal muscle was bisected to expose the skull, and the MCA was exposed by means of burr-hole craniotomy carried out by using a surgical drill. A thin layer of bone was preserved to protect the dura mater and the cortical surface against mechanical damage and thermal injury, while the remaining bone was gently removed. The MCA was occluded by electrocoagulation, and the muscle and then the skin incision were sutured. A rectal temperature probe connected to a heating pad was used to maintain body temperature at 37°C throughout the surgical period. After surgery, the mice did not receive anti-inflammatory drugs or antibiotics, were placed in an incubator (compact incubator, Thermo Scientific, AHSI, Bernaggio, MI, Italy) at 37°C for 2, and then placed back into their home cages. The functional deficit was assessed with the paw placement test before ischemia and after 4 and 24 h by an operator who was unaware of the genotype. The animals were killed 24 h after ischemia, and their brains were processed for histological or mRNA analysis.

### Paw Placement Test

Paw placement test provides information on the tactile/proprioceptive response to limb stimulation. The animals were placed with all paws on a surface in horizontal position, and the head was held at 45° angle so that visual stimulation was prevented. The paws to be tested were pushed along the edge in order to lose contact with the table surface. The ability of the animals to place the limb back onto the table surface when the mice was moved toward the edge was evaluated. A score was used as follows: 1—prompt placement of the limb onto the table, 0.5—incomplete or delayed placing of the limb; and 0—no placing with the extension of the limb and paw (29).

### Histology

The mice were sacrificed 24 h after MCA occlusion, and the brains were fixed in Carnoy's solution, embedded in paraffin, and sectioned at 10 μm. The sections were deparaffinized and processed for staining with thionin (Nissl staining) for histological assessment of neuronal degeneration. The analysis was carried out on sections regularly spaced every 550 μm through the extension of the ischemic region. This space interval corresponds to a standardized procedure, allowing one to measure the ischemic brain volume based on 10 slides. The infarct area was outlined at a magnification of ×2.5, and it was quantified using Scion Image software (NIH, Bethesda, MD, USA). Then, the volume was calculated by integrating the cross-sectional area of damage on each stained section and the distance between them (20, 30).

### Immunohistochemistry

Brain sections were deparaffinized, after antigen retrieval, in citrate buffer (10 mM sodium citrate, pH 6.0) for 30 min. The sections were pretreated with 0.3% H_2_O_2_ for 10 min to block endogenous peroxide activity after incubation with 6% normal serum and 0.1% Triton-X100 in PBS for 2 h. The sections were incubated with the following primary antibodies: anti-Cox2 (1:200, Abcam, Cambridge, UK) or anti-Iba1 (1:1,000, Wako Chemicals, Osaka, Japan). The samples were rinsed and incubated for 2 h at room temperature with secondary biotinylated anti-rabbit horse antibodies (1:200, Vector Laboratories, Burlingame, CA) followed by streptavidin alexa fluor 488 (1:200, Molecular Probes, Carlsbad, CA). Finally, the samples were mounted with Hard Set Vectashield with Dapi (Vector Laboratories, Burlingame, CA). Iba1 immunostaining was detected with a Zeiss 780 confocal laser scanning microscope. Cox-2 immunostaining was detected with a Zeiss Axio-photo 2 optic microscope. Cell counting was performed in three coronal sections for mouse at ×20 magnification.

### RNA Purification, cDNA Synthesis, and Real-Time qPCR

The mice were killed 24 h after MCA occlusion, and the brains were quickly removed. The brains were cut with a matrix, and a 3-mm region along the rostro-caudal axis from 1.54 mm anterior to 1.58 mm posterior to the bregma was sliced. Each slice was further punched to dissect a square region (1 × 1.3 mm) corresponding to the ischemic core, the dorsomedial portion of the peri-infarct region or the corresponding region of the contralateral side (highlighted in **Figures 2A**, **4A**). The samples were immediately frozen on liquid nitrogen and stored at −80°C. Total RNA was extracted using Nucleazol reagent and the NucleoSpin RNA Set for NucleoZol (Macherey-Nagel, Hoerdt, France) according to the manufacturer's instruction. RNA quantity and quality were determined using the NanodropTM apparatus (Thermofisher Scientific, Walthman, MA), and 0.5 μg of total RNA was used to perform a reverse transcription (IscriptTM cDNA synthesis kit, Bio-Rad, Marnes-la-Coquette, France). The qPCR measurements were performed in triplicate using SYBR Green Super-mix (Bio-Rad, Marnes-la-Coquette, France). The reaction conditions were as follows: 98°C for 30 s (polymerase activation), followed by 40 cycles at 95°C for 5 s, 60°C for 10 s, and 72°C for 10 s. The primers were designed using Primer3Plus software (Table 1). A melting curve analysis was used to assess the specificity of the selected primers, and the results were quantified using relative standard curve methods. The target gene relative expression in the dorsomedial peri-infarct regions and their respective contralateral sides was calculated after normalization to the ribosomal protein L13 (Rpl13) reference gene. The following reference genes—Rpl13, Hmbs, and Rn18s—were assessed in the core area and its respective contralateral side. Data are expressed as mean ± SEM and normalized to the mGlu3^+/+^ contralateral group.

**Table 1:** Gene primers.

**Gene**	**Forward**	**Reverse**
Arg1	GTGAAGAACCCACGGTCTGT	GCCAGAGATGCTTCCAACTG
Cd86	GAGCGGGATAGTAACGCTGA	GGCTCTCACTGCCTTCACTC
Gdnf	GCACCCCCGATTTTTGC	AGCTGCCAGCCCAGAGAATT
Hmbs	TCCCTGTTCAGCAAGAAGATG	GGATGTTCTTGGCTCCTTTG
Il1b	GAAGATGGAAAAACGGTTTG	GTACCAGTTGGGGAACTCTGC
Il6	CAAAGCCAGAGTCCTTCAGA	GCCACTCCTTCTGTGACTCC
Il4ra	GGATAAGCAGACCCGAAGC	ACTCTGGAGAGACTTGGTTGG
Mrc1	CTTCGGGCCTTTGGAATAAT	TAGAAGAGCCCTTGGGTTGA
Ptgs2	TCATTCACCAGACAGATTGCT	AAGCGTTTGCGGTACTCATT
Rn18s	CGGTACAGTGAAACTGCGAAT	CCGTGGGCATGTATTAGCTC
Rpl13	ACAGCCACTCTGGAGGAGAA	GAGTCCGTTGGTCTTGAGGA
Tnfa	GCCTCTTCTCATTCCTGCTT	AGGGTCTGGGCCATAGAACT
Tgfb	TGATACGCCTGAGTGGCTGTCT	CACAAGAGCAGTGAGCGCTGAA
Socs3	CGTTGACAGTCTTCCGACAA	TATTCTGGGGGCGAGAAGAT

### Statistical Analysis

Statistical analysis of all data was performed using GraphPad PRISM software, version 8.0. In all experiments, data are presented as means ± standard error of the mean (SEM), and *p* < 0.05 was considered significant. A two-tailed unpaired Student's *t*-test was performed for two-group comparisons in infarct volume. The mRNA data were normalized to the wild-type contralateral group and analyzed using two-way ANOVA, followed by Fisher's *post hoc* comparison tests. For behavioral analysis, the non-parametric Mann–Whitney *U* test and Friedman ANOVA were performed.

## Results

### Genetic Deletion of mGlu3 Receptors Enhanced Infarct Size and Neuroinflammation in CD1 Mice Undergoing Permanent MCA Occlusion

**Figure 1:**
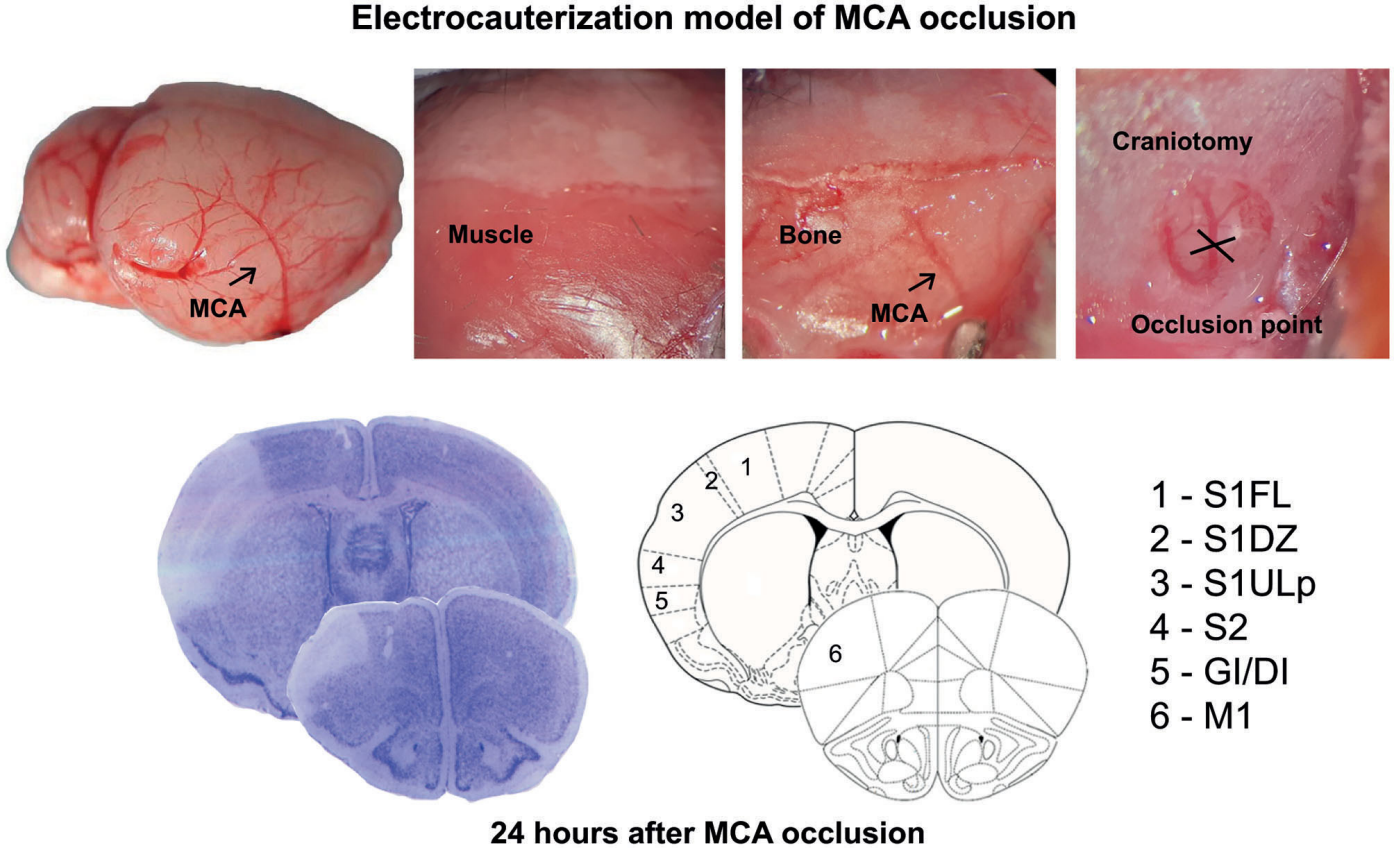
Schematic representation of the permanent occlusion of the middle cerebral artery and regional topography of ischemic infarct.

**Figure 2:**
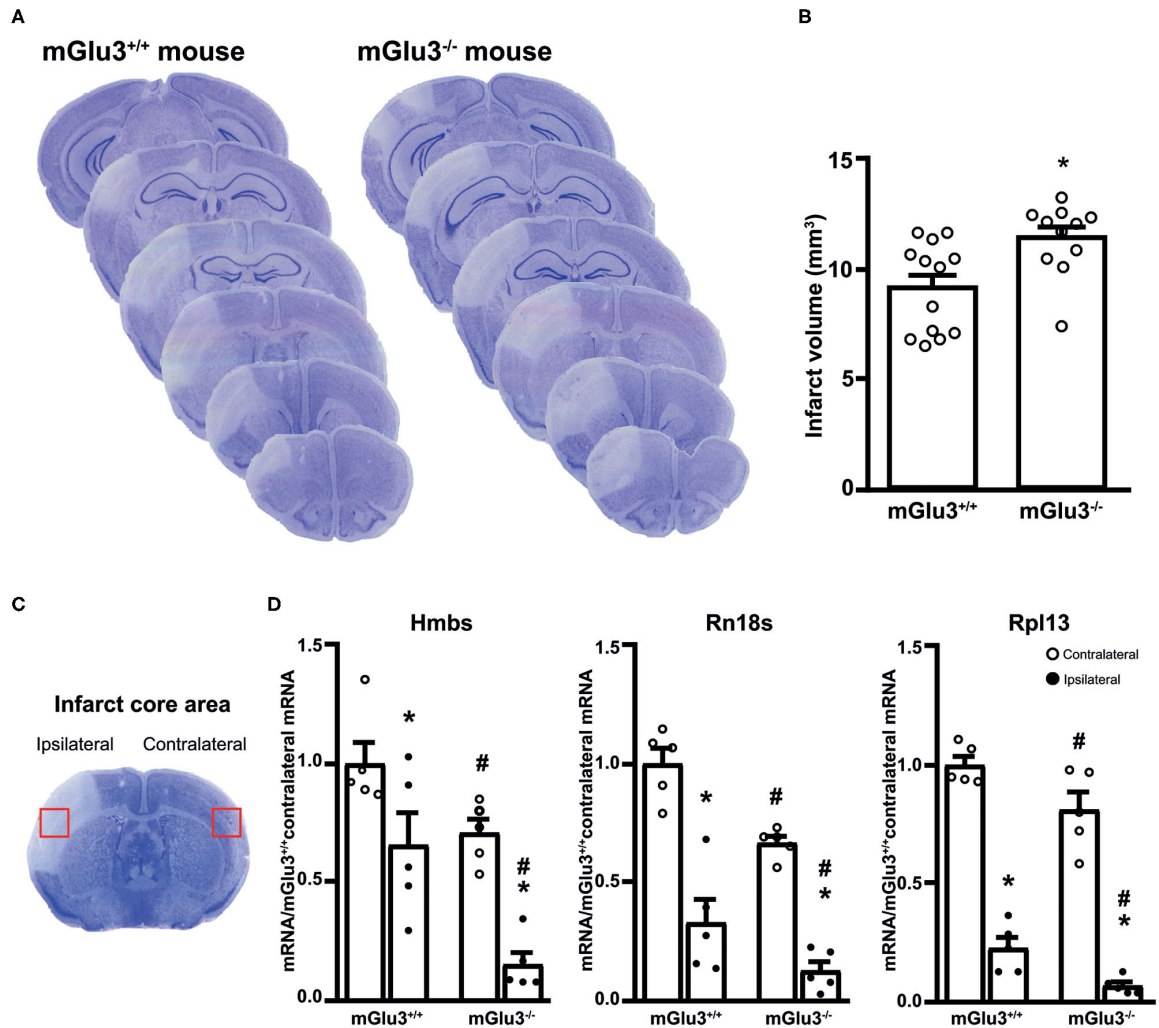
Infarct size and expression of housekeeping genes in the infarct core of CD1 mGlu3^+/+^ and mGlu3^−/−^ mice subjected to permanent middle cerebral artery (MCA) occlusion. Nissl staining of sequential coronal brain sections of mGlu3^+/+^ and mGlu3^−/−^ mice 24 h following MCA occlusion is shown in **(A)**. Quantification of the infarct volume is shown in **(B)**, where values are means ± S.E.M. of 11–13 mice per group. *p < 0.05 vs. mGlu3^+/+^ mice (Student's *t* test; *t*_x_ = 2.967). The infarct core region dissected for measurements of housekeeping genes is indicated in **(C)**. Hmbs, Rn18s, and Rpl13 mRNA levels in the ipsilaeral and contralateral sides of the two genotypes are shown in **(D)**, where values are means ± S.E.M. of five determinations. *p* < 0.05 (two-way ANOVA + Fisher's least significant difference) vs. the contralateral side of the same genotype (*) or the corresponding side of mGlu3^+/+^ mice (#). Hmbs: genotype, *F*_1,16_ = 19.34, *p* = 0.0004; side, *F*_1,16_ = 24.67, *p* = 0.0001; interaction, *F*_1,16_ = 1.396, *p* = 0.2546; Rn18s: genotype, *F*_1,16_ = 17.48, *p* = 0.0007; side, *F*_1,16_ = 88.85, *p* < 0.0001; interaction, *F*_1,16_ = 1.086, *p* = 0.3167; Rpl13: genotype, *F*_1,16_ = 12.73, *p* = 0.0026; side, *F*_1,16_ = 243.3, *p* > 0.0001; interaction, *F*_1,16_ = 0.0936, *p* = 0.7636.

Ischemic infarct caused by permanent distal MCA occlusion in CD1 mice included the primary motor cortex (M1), the forelimb (S1FL), dysgranular (S1DZ), and upper lip (S1ULp) regions of the primary somatosensory cortex, the secondary somatosensory cortex (S2), and the granular/dysgranular insular cortex (GI/DI) (Figure 1). The ischemic infarct was detectable as early as 2 h after MCA occlusion and reached its maximal size at 24 h. The extent of the infarct volume, evaluated by Nissl staining at 24 h after MCA occlusion, was greater in mGlu3^−/−^ mice as compared to their mGlu3^+/+^ counterparts (Figures 2A,B). We measured the transcripts of three housekeeping genes (Hmbs, Rn18s, and Rpl13) in a small region of the ischemic core (highlighted in Figure 2C) as an unbiased method to detect cell damage in response to permanent ischemia. In mGlu3^+/+^ mice, the mRNA levels of the three transcripts were significantly reduced in the ischemic core with respect to the corresponding region of the contralateral hemisphere. The reduction was much greater in the ischemic core of mGlu3^−/−^ mice as compared to the respective contralateral site and also to the ischemic core of mGlu3^+/+^ mice (Figure 2D). Interestingly, gene expression was also lower in the contralateral site of mGlu3^−/−^ mice compared to the contralateral site of mGlu3^+/+^ mice (Figure 2D).

**Figure 3:**
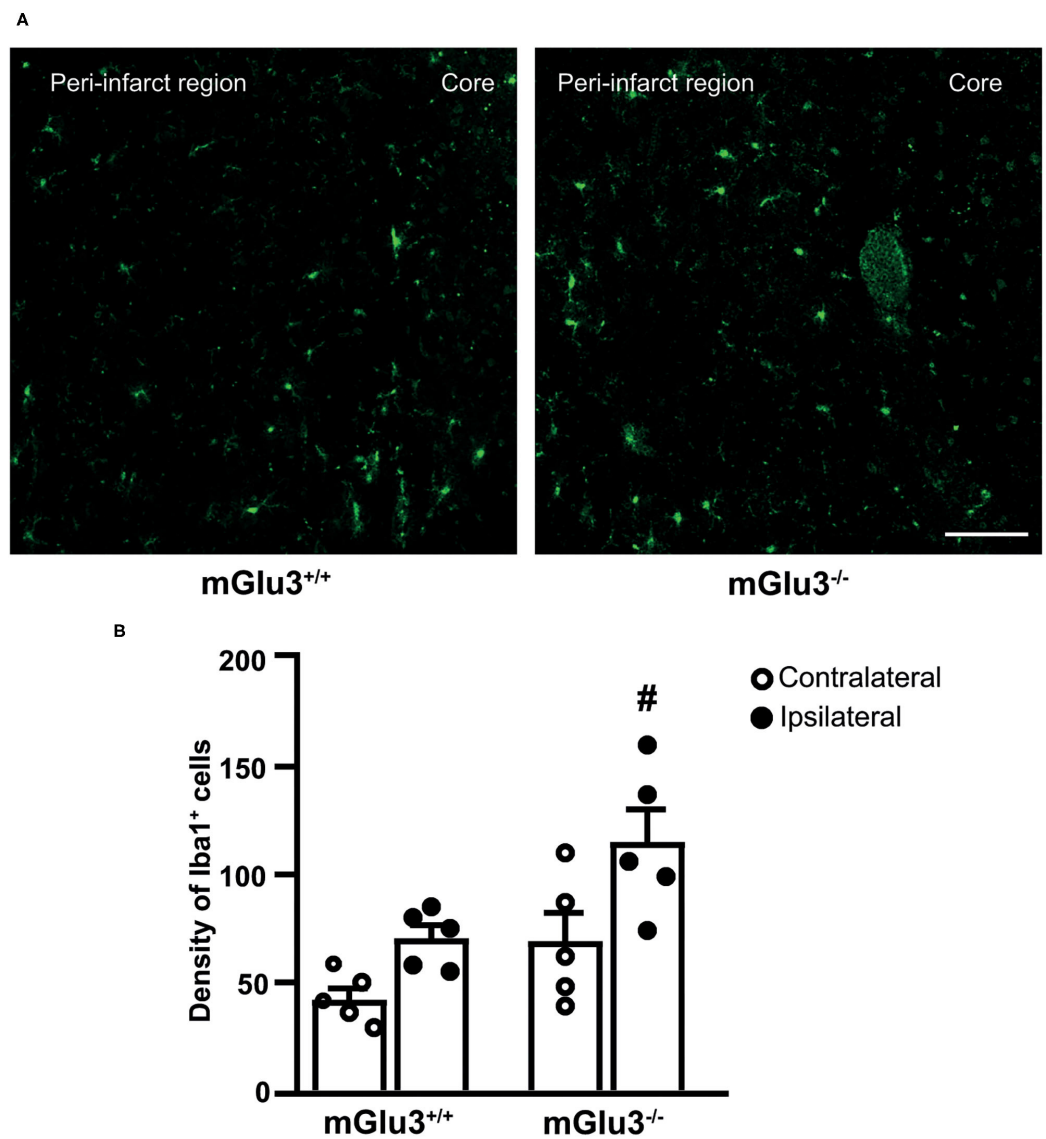
Iba1 immunostaining in the peri-infarct region and the corresponding contralateral region of CD1 mGlu3^+/+^ and mGlu3^−/−^ mice subjected to middle cerebral artery occlusion. The density of Iba1^+^ cells was measured in three sections of the peri-infarct and contralateral regions of the two genotypes. Representative images are shown in **(A)**. Values are means ± S.E.M. from five mice per group. #*p* < 0.05 vs. the peri-infarct region of mGlu3^+/+^ mice (two-way ANOVA + Fisher's least significant difference). Genotype, *F*_1,16_ = 11.22, *p* = 0.0041; side, *F*_1,16_ = 12.11, *p* = 0.0031; interaction, *F*_1,16_ = 0.6590, *p* = 0.4288. Scale bar = 50 mm.

**Figure 4:**
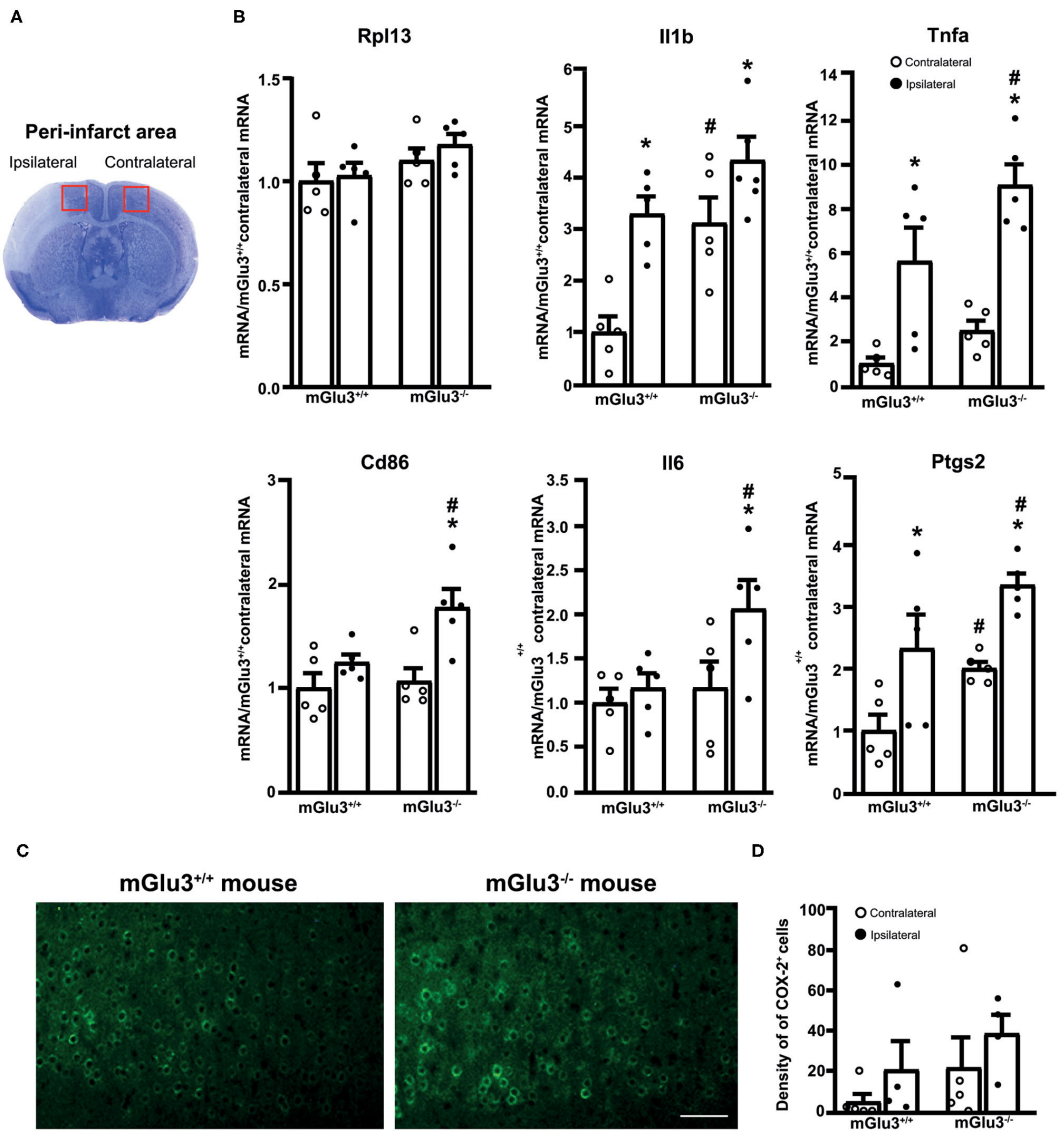
Expression of pro-inflammatory genes in the peri-infarct region and the corresponding contralateral region of CD1 mGlu3^+/+^ and mGlu3^−/−^ mice subjected to middle cerebral artery (MCA) occlusion. The anatomical location of the dissected dorsomedial peri-infarct region and the corresponding contralateral region is shown in **(A)**. The mRNA levels of the selected housekeeping and pro-inflammatory genes of the ipsilateral and contralateral sides of mGlu3^+/+^ and mGlu3^−/−^ mice subjected to MCA occlusion is shown in **(B)**. Values are means ± S.E.M. of five determinations. *p* < 0.05 (two-way ANOVA + Fisher's least significant difference) vs. the contralateral side of the same genotype (*) or the corresponding side of mGlu3^+/+^ mice (#). Rpl13: genotype, *F*_1,16_ = 3.652, *p* = 0.0741; side, *F*_1,16_ = 0.6079, *p* = 0.4470; interaction, *F*_1,16_ = 0.1461, *p* = 0.7074; Il1b: genotype, *F*_1,16_ = 15.26, *p* = 0.0013; side, *F*_1,16_ = 18.79, *p* = 0.0005; interaction, *F*_1,16_ = 1.756, *p* = 0.2038; Tnfa: genotype, *F*_1,16_ = 7.016, *p* = 0.017; side, *F*_1,16_ = 36.5, *p* > 0.0001; interaction, *F*_1,16_ = 1.11, *p* = 0.3078; Cd86: genotype, *F*_1,16_ = 4.826, *p* = 0.0432; side, *F*_1,16_ = 12.62, *p* = 0.0027; interaction, *F*_1,16_ = 0.0012, *p* = 0.97; Il6: genotype, *F*_1,16_ = 4.618, *p* = 0.0473; side, *F*_1,16_ = 4.584, *p* = 0.048; interaction, *F*_1,16_ = 2.142, *p* = 0.1627; Ptgs2: genotype, *F*_1,16_ = 10.09, *p* = 0.0012; side, *F*_1,16_ = 17.54, *p* = 0.0007; interaction, *F*_1,16_ = 0.0012, *p* = 0.97. Representative COX-2 immunostaining in the peri-infarct regions of the mGlu3^+/+^ and mGlu3^−/−^ mice is shown in **(C)**. The density of COX-2-expressing cells in three sections of the peri-infarct and contralateral regions of the two genotypes is shown in **(D)**, where values are means ± S.E.M. from four mice per group. Statistical analysis was performed by two-way ANOVA + Fisher's least significant difference. Genotype, *F*_1,14_ = 2.284, *p* = 0.1529; side, *F*_1,14_ = 2.006, *p* = 0.1785; interaction, *F*_1,14_ = 0.001865, *p* = 0.966. Scale bar = 25 μm.

To examine whether the genetic deletion of mGlu3 receptors amplified neuroinflammation in response to MCA occlusion, we first performed immunofluorescent staining of the microglia/macrophage marker Iba1 (31) in the peri-infarct region and the corresponding contralateral region (Figure 3A). The density of Iba1^+^ cells (measured in three microscopic sections) was greater in the peri-infarct regions of mGlu3^−/−^ mice as compared to the peri-infarct region of mGlu3^+/+^ mice (Figure 3B). The mGlu3^−/−^ mice also showed a trend to an increased density of Iba1^+^ cells in the contralateral region (Figure 3B). We then measured the transcripts of a number of pro-inflammatory, immune-modulatory, and anti-inflammatory genes in the dorsomedial peri-infarct and contralateral regions (dissected as shown in Figure 4A). There was no difference in the expression of the housekeeping gene Rpl13 between the ipsilateral and contralateral sides in the two genotypes, suggesting the lack of cell death in the peri-infarct region (Figure 4B). In mGlu3^+/+^ mice, the transcripts of Il1b, Ptgs2, and Tnfa genes encoding the proinflammatory cytokines, IL-1β, COX-2, and TNF-α, showed a significant increase in the peri-infarct region compared to the corresponding contralateral region, whereas the transcripts of Cd86 and Il6, encoding CD86 and IL-6, respectively, were unchanged (Figure 4B). In contrast, the expression of all five pro-inflammatory genes was largely increased in the peri-infarct region of mGlu3^−/−^ mice, compared to the corresponding contralateral region (Figure 3B). Remarkably, the Ptgs2, Tnfa, Cd86, and Il6 mRNA levels were significantly higher in the peri-infarct region of mGlu3^−/−^ mice with respect to the peri-infarct region of mGlu3^+/+^ mice, and a trend to increase was also observed for the transcript encoding IL-1β (Figure 4B). Changes in COX-2 immunostaining paralleled the changes of the Ptgs2 transcript (greater density of COX-2^+^ cells in both sides of mGlu3^−/−^ mice), although the differences were not statistically significant (Figures 4C,D).

**Figure 5:**
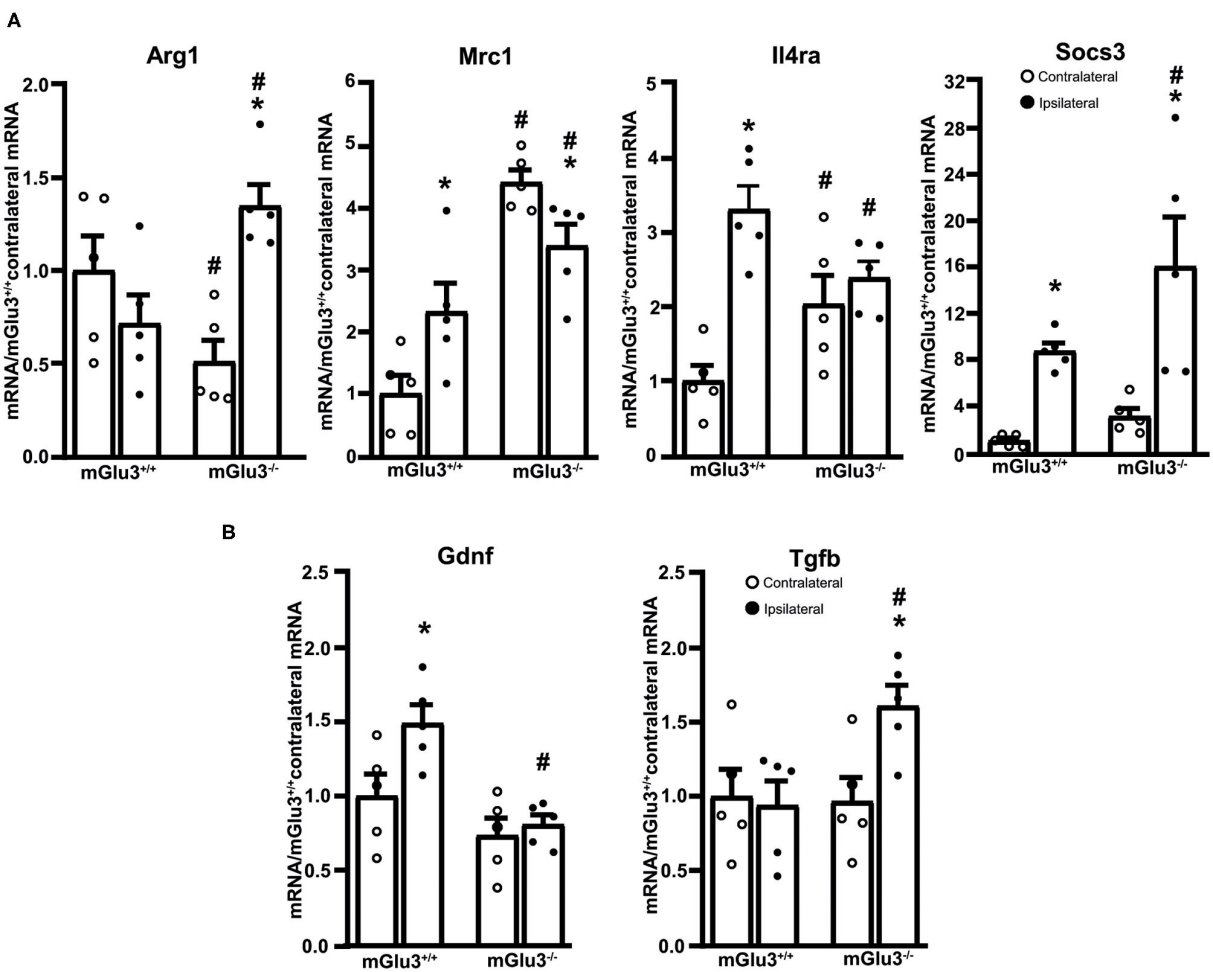
Expression of immunoregulatory, anti-inflammatory, and neuroprotective genes in the peri-infarct region of CD1 mGlu3^+/+^ and mGlu3^−/−^ mice subjected to middle cerebral artery (MCA) occlusion. The mRNA levels of the selected immunoregulatory and anti-infammatory genes of the ipsilateral and contralateral sides of wild-type and mGlu3^−/−^ mice subjected to MCA occlusion is shown in **(A)**. The mRNA levels of the genes encoding GDNF and TGF-β are shown in **(B)**. Values are means ± S.E.M. of five determinations. *p* < 0.05 (two-way ANOVA + Fisher's LSD) vs. the contralateral side of the same genotype (*) or the corresponding side of mGlu3^+/+^ mice (#). Arg1: genotype, *F*_1,16_ = 0.2441, *p* = 0.628; side, *F*_1,16_ = 3.639, *p* = 0.0746; interaction, *F*_1,16_ = 14.98, *p* = 0.0014; Mrc1: genotype, *F*_1,16_ = 43.5, *p* < 0.0001; side, *F*_1,16_ = 0.1896, *p* = 0.6691; interaction, *F*_1,16_ = 11.97, *p* = 0.0032; Il4ra: genotype, *F*_1,16_ = 0.037, *p* = 0.848; side, *F*_1,16_ = 20.81, *p* = 0.0003; interaction, *F*_1,16_ = 11.19, *p* = 0.0041; Socs3: genotype, *F*_1,16_ = 4.619, *p* = 0.0473; side, *F*_1,16_ = 22.29, *p* = 0.0002; interaction, *F*_1,16_ = 1.413, *p* = 0.252; Gdnf: genotype, *F*_1,16_ = 16.19, *p* = 0.001; side, *F*_1,16_ = 5.731, *p* = 0.029; interaction, *F*_1,16_ = 3.118, p = 0.0965; Tgfb: genotype, *F*_1,16_ = 3.771, *p* = 0.0472; side, *F*_1,16_ = 3.18, *p* = 0.093; interaction, *F*_1,16_ = 4.621, *p* = 0.0472.

The expression of three immunomodulatory genes (Mrc1, Il4ra, and Socs3, encoding MRC-1, IL4Ra, and SOCS3, respectively) largely increased in the peri-infarct region of mGlu3^+/+^ mice, whereas only the transcript encoding SOCS3 increased in the peri-infarct region of mGlu3^−/−^ mice as compared to the contralateral corresponding region (Figure 5A). Mrc1 expression was lower in the ipsilateral side of mGlu3^−/−^ mice as opposed to what was found in mGlu3^+/+^ mice. Interestingly, however, the Mrc1 mRNA levels were significantly higher in both sides of mGlu3^−/−^ mice with respect to the corresponding regions of mGlu3^+/+^ mice (Figure 5A). The Socs3 mRNA levels increase to a greater extent in the peri-infarct region of mGlu3^−/−^ mice than in the peri-infarct region of mGlu3^+/+^ mice (Figure 5A).

The expression of the anti-inflammatory gene Arg1 (encoding arginase-1) did not differ between the ipsilateral and contralateral sides of mGlu3^+/+^ mice after MCA occlusion. In mGlu3^−/−^ mice, the Arg1 mRNA levels showed a large increase in the ipsilateral side. In addition, the levels were lower in the contralateral side and higher in the ipsilateral side with respect to the corresponding regions of mGlu3^+/+^ mice (Figure 5A).

Using the same extracts from the peri-infarct region, we extended the analysis to two trophic factors (GDNF and TGF-β), which are under the control of mGlu3 receptors. Interestingly, the transcript encoding GDNF was exclusively increased in the peri-infarct region of mGlu3^+/+^ mice, whereas the transcript encoding TGF-β was increased in the peri-infarct region of mGlu3^−/−^ mice (Figure 5B).

### Genetic Deletion of mGlu3 Receptors Enhanced Infarct Size and Worsened the Impairment of Motor Response to Tactile/Proprioceptive Stimuli in C57Black Mice Subjected to Permanent MCA Occlusion

We used C57Black mice to confirm the effect of mGlu3 receptor deletion in another strain of mice and to extend the study to the assessment of behavioral impairment induced by ischemia with the paw placement test. This test evaluates the tactile/proprioceptive response of the animal and is widely used in rodent models of brain ischemia (29). This test could not be applied to CD1 mice because of their suboptimal performance under normal conditions.

**Figure 6:**
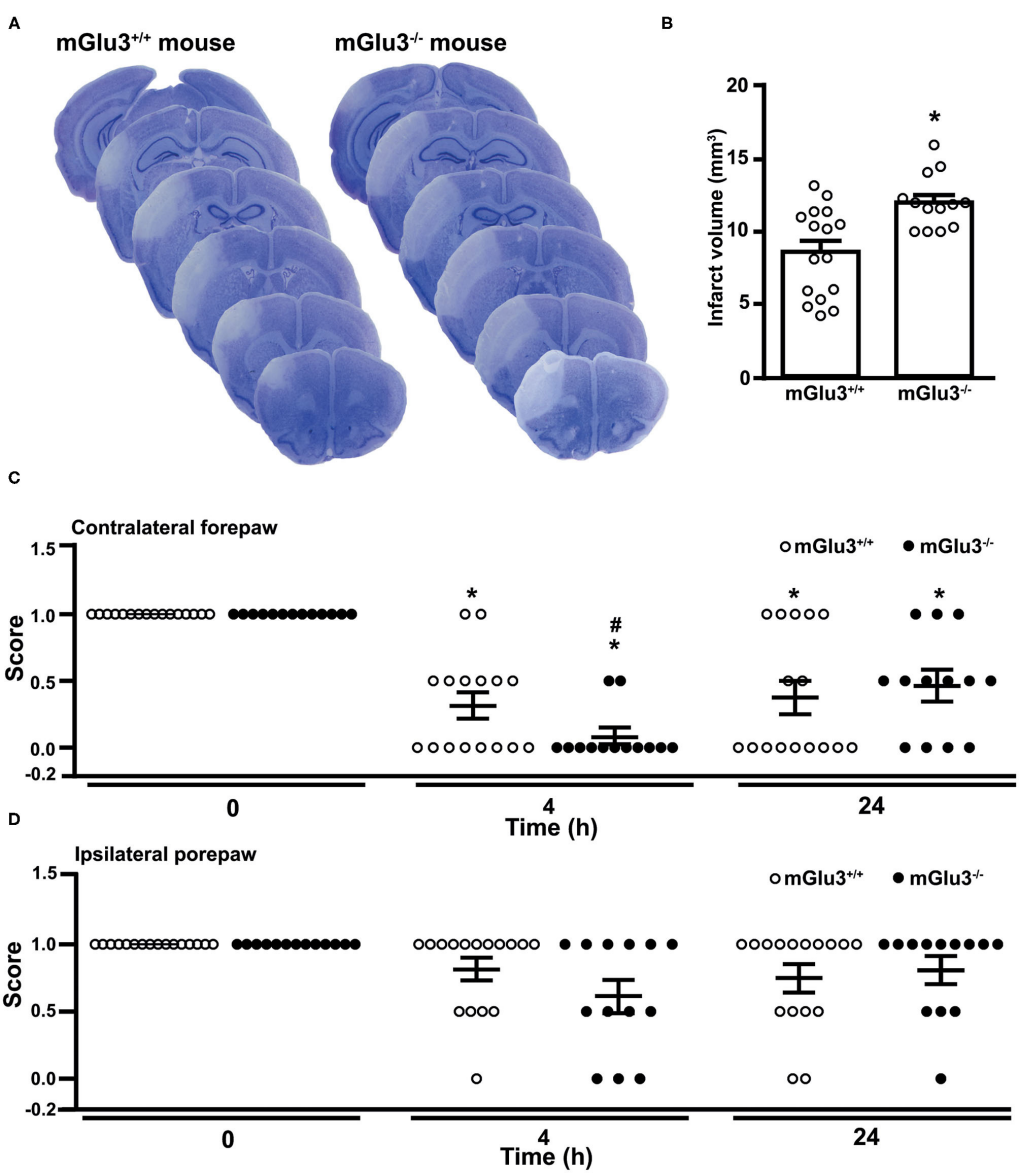
Infarct size and performance in the paw placement test of C57Black mGlu3^+/+^ and mGlu3^−/−^ mice subjected to permanent middle cerebral artery (MCA) occlusion. Nissl staining of sequential coronal brain sections of mGlu3^+/+^ and mGlu3^−/−^ mice 24 h following MCA occlusion is shown in **(A)**. Quantification of the infarct volume is shown in **(B)**, where values are means ± S.E.M. of 13–16 mice per group. *p < 0.05 vs. mGlu3^+/+^ mice (Student's *t* test; *t*_x_ = 3.501). The paw placement scores of the contralateral and ipsilateral forepaws are shown in **(C,D)**, respectively. Means ± S.E.M. are indicated. Contralateral side: *p < 0.05 vs. the corresponding basal values (time 0) (Friedman non-parametric ANOVA test + Dunn's; Friedman statistical value = 20.65) or ^#^*p* < 0.05 vs. values of mGlu3^+/+^ mice at 4 h (one-tailed Mann–Whitney non-parametric test).

Similarly to what was found in CD1 mice, the infarct size was significantly greater in mGlu3^−/−^ mice of C57Black strain subjected to permanent MCA occlusion as compared to their mGlu3^+/+^ counterparts (Figures 6A,B). Remarkably, the extent of the increase in infarct size as measured in ischemic C57Black mice lacking mGlu3 receptors was identical to that seen in CD1 mGlu3^−/−^ mice (Figures 2B, 6B).

The paw placement test was performed before ischemia, both at 4 and 24 h following MCA occlusion. At 4 h, the impairment of the proprioceptive/tactile response of the forepaw contralateral to MCA occlusion was significantly greater in mGlu3^−/−^ than in mGlu3^+/+^ mice (Figure 6C). At 24 h, there was no difference between the two genotypes because of a partial recovery of mGlu3^−/−^ mice (Figure 6C). There were only small and non-significant changes in the ipsilateral forepaw placement in the two time points following MCA occlusion, although a trend to reduction in the disability score was observed in mGlu3^−/−^ mice at 4 h (Figure 6D).

## Discussion

The role of mGlu receptors in mechanisms of ischemic neuronal cell death has been the subject of extensive investigation. Pharmacological blockade of mGlu1 receptors was shown to be neuroprotective in hippocampal slices subjected to a paradigm of oxygen/glucose deprivation and in models of transient global ischemia (32, 33). Conversely, the activation of mGlu1 receptors was found to mediate ischemic tolerance induced by “ischemic preconditioning” in hippocampal slice preparations (34). However, these findings have not been translated into effective treatment strategies in stroke because mGlu1 receptors may display neurotoxic and neuroprotective functions depending on the cell context and the paradigm of neuronal death (35, 36). The use of mGlu5 receptor ligands in stroke models has generated conflicting results. Early studies showed that both mGlu5 receptor agonists and antagonists reduced infarct size in a rat intraluminal filament model of transient MCA occlusion (37), whereas pharmacological activation of mGlu5 receptors was not protective in the endothelin-1 rat model of focal ischemia (38). More recent findings showed that selective pharmacological blockade of mGlu5 receptors reduced microglial activation and neuronal death induced by acute intracerebral hemorrhage (39). The mGlu4 receptors are also potential drug targets for neuroprotection in ischemic stroke as shown by the evidence that mGlu4 receptor activation reduced infarct size in ischemic mice and rats, whereas brain damage was amplified in mGlu4^−/−^ mice (40). Activating mGlu4 receptors might also restrain immune reactive mechanisms in stroke (6) by promoting immune tolerance (41).

Our data offer the first evidence for a neuroprotective activity of mGlu3 receptors against focal brain ischemia in two different strains of mice. mGlu2 and mGlu3 receptors show a high degree of primary sequence homology, are both coupled to G_i/o_ proteins in heterologous expression systems, and share some functional properties including, for example, the ability to restrain neurotransmitter release (9). However, using both genetic and selective pharmacological tools, we were able to demonstrate that activation of mGlu2 receptors amplifies brain damage in rodent models of global and focal ischemia (19, 20). Thus, a comparison between present findings and previous data reveals that mGlu3 and mGlu2 receptors have an opposite impact on vulnerability to ischemic brain damage. There are at least two functions of mGlu3 receptors that account for this difference: the anti-inflammatory action in microglia (14, 16, 42) and the stimulation of TGF-β, and GDNF production in astrocytes and neurons, respectively (21–23, 43, 44). We have found that gestational low-protein diet (LPD) combined with IL-1β injection in rat pups (modeling intrauterine growth restriction and perinatal brain inflammation in human neonates) was associated with a selective down-regulation of mGlu3 receptors in microglia. In addition, microglia reactivity to inflammatory challenge induced by LPD/IL-1β was reduced by the pharmacological activation of mGlu3 receptors, whereas pharmacological blockade or the genetic deletion of mGlu3 receptors induced an inflammatory phenotype in microglia (16). In contrast, mGlu2 receptor activation promotes a pro-inflammatory and neurotoxic phenotype in microglia (14, 15). The large increase in the expression of pro-inflammatory genes found in the peri-infarct region of mGlu3^−/−^ mice strengthens the hypothesis that mGlu3 receptors are key regulators of microglial function and act to restrain neuroinflammation. This mechanism may critically shape neuronal vulnerability to focal ischemia because neuroinflammation caused by the activation of resident microglia and infiltration of peripheral monocytes is consistently associated with ischemic stroke, leading to secondary injury cascade and neuronal death (45–49).

The reduced expression of Mrc1 (vs. the contralateral side) and Il4ra (vs. mGlu3^+/+^ mice) found in the peri-infarct region of mGlu3^−/−^ mice is consistent with a putative anti-inflammatory action of mGlu3 receptors. Mrc1 encodes mannose receptor 1, also referred to as CD206, which is involved in the mechanisms of pinocytosis and phagocytosis and is associated with the anti-inflammatory and neuroprotective phenotype in microglia (50–53). Il4ra encodes the α-subunit of the IL-4 receptor, which mediates the anti-inflammatory effect of IL-4 in macrophages and microglia (54, 55). Alternatively, the increased expression of Arg1 was found exclusively in the peri-infarct region of mGlu3^−/−^ mice. Arg1 encodes for arginase 1, the enzyme that converts arginine into L-ornithine and is considered a marker for the anti-inflammatory phenotype of microglia. Accordingly, L-arginine is the same substrate for both nitric oxide synthase (NOS) and arginase 1, and the Arg1 outcompetes inducible NOS to reduce the production of nitric oxide (56). The STAT6 (type-6 Signal Transduction and Activator of Transcription)/Arg1 pathway promotes microglia/macrophase efferocytosis (phagocytosis of dying/dead cells), thus facilitating the resolution of inflammation and preventing further cell death in mice subjected to MCA occlusion (57, 58). The increased Arg1 expression detected in the peri-infarct region of mGlu3^−/−^ mice might reflect a compensatory mechanism aimed at restraining the neuroinflammation, mediated by M2 microglia polarization (57, 58). The increase in SOCS3 transcript seen in mGlu3^−/−^ mice might be a component of this compensatory mechanism because SOCS3 inhibits cytokine receptor signaling, although SOCS3 has pleiotrophic activities and might be detrimental for cell survival *via* the induction of the pro-apoptotic metabolite, ceramide (59).

Studies performed in cell cultures and living mice have shown that the pharmacological activation of mGlu3 receptors stimulates the production of TGF-β in astrocytes (21–23) and GDNF in neurons (43, 44). Both GDNF and TGF-β are neuroprotective, and GDNF requires TGF-β for its neuroprotective action (60). The reduced GDNF transcript levels found in the peri-infarct region of mGlu3^−/−^ mice are in line with the evidence that activation of mGlu3 receptors enhances GDNF production in the CNS (43, 44). Lowered GDNF levels might contribute to the increased infarct size in mGlu3^−/−^ mice because GDNF is known to exert a neuroprotective activity.

In contrast, the increase in TGF-β mRNA levels found in the peri-infarct region of mGlu3^−/−^ mice was unexpected because mGlu3 receptor activation is known to stimulate TGF-β production in astrocytes (17). One explanation is that, other cells, such as macrophages and microglia, are the source of TGF-β in the peri-infarct region of mGlu3^−/−^ mice. How changes in TGF-β in gene transcripts contribute to mechanisms of neurodegeneration/neuroprotection in the peri-infarct region is unknown. However, a causal relatioship may exist between the increase in TGF-β and the reduction in Mrc1 in the peri-infarct region of mGlu3^−/−^ mice because TGF-β negatively modulates Mrc1 expression (61, 62).

Behavioral analysis performed in C57Black mice showed a greater defect in the paw placement test in mGlu3^−/−^ mice at short times (4 h) following MCA occlusion. This might reflect the early brain damage and hyperinflammation caused by the lack of mGlu3 receptors. However, the performance in the paw placement test partially recovered, and there was no difference between mGlu3^+/+^ and mGlu3^−/−^ mice at 24 h. An increased glutamate release caused by the lack of presynaptic mGlu3 receptors might contribute to excitotoxic neuronal death but, on the other side, might facilitate functional recovery by inducing mechanisms of activity-dependent synaptic plasticity. This hypothesis warrants further investigation.

In conclusion, our data suggest that the endogenous activation of mGlu3 receptors display a protective activity against ischemic brain damage and associated neuroinflammation and lays the groundwork for the use of subtype-selective mGlu3 receptor agonists or positive allosteric modulators (PAM) in experimental animal models of stroke. A selective mGlu3 receptor agonist is already available (63), and mGlu3 receptor PAMs are under development (64). These are putative drug candidates as neuroprotectants in ischemic stroke.

## Data Availability Statement

The raw data supporting the conclusions of this article will be made available by the authors, without undue reservation.

## Ethics Statement

The animal study was reviewed and approved by Neuromed Institutional Animal Care and Use Committee and the Italian Ministry of Health. Written informed consent was obtained from the owners for the participation of their animals in this study.

## Author Contributions

FM: conceptualization, investigation, and formal analysis. MZ, GM, JP, and TI: investigation. VB, GB, JM, OB, and FN: supervision, data curation, and writing. All authors contributed to the article and approved the submitted version.

## Conflict of Interest

The authors declare that the research was conducted in the absence of any commercial or financial relationships that could be construed as a potential conflict of interest.
